# Supplementary material for: 3,6′- and 1,6′-Dithiopomalidomide Mitigate Ischemic Stroke in Rats and Blunt Inflammation
Source: Pharmaceutics. 2022 Apr 27;14(5):950. doi: 10.3390/pharmaceutics14050950 (PMC9146426; doi:10.3390/pharmaceutics14050950)
Supplement: Supplementary file 1 [file pharmaceutics-14-00950-s001.zip › pharmaceutics-1643298-supplementary.pdf]

Supplemental Materials

3,6′- and 1,6′-Dithiopomalidomide Mitigate Ischemic Stroke in the Rat and Blunt Systemic and Brain Inflammation

1. Drug Synthesis

1.1. 3,6′-DP: Following the procedure of Scerba et al.,[1] Pomalidomide (273mg, 1.0mmol, 1.0eq) and P_4S_{10}-pyridine complex (570mg, 1.5mmol, 1.5eq) were charged into a 100mL round bottom flask along with a stir bar. The system was affixed with a reflux condenser, and the vessel placed under N_2. Then, 27mL of 1,4-dioxane was injected and the mixture was stirred and heated to 100°C. After workup and prep-HPLC purification, pure 3,6′-DT was isolated as a bright orange powder. 96mg, 31% yield. ^{1}H NMR (400 MHz, DMSO-d_6) δ 12.63 (s, 1H), 7.65 (bs, 2H), 7.50 – 7.40 (m, 1H), 7.18 (d, J = 8.5 Hz, 1H), 7.03 (bs, 1H), 5.64-5.40 (bm 1H*), 3.26-2.78 (b, 2.5H*), 2.02 (bm, 1H). Note that the starred * signals are very broad and proved difficult to resolve even after increasing relaxation delays and scans. It is for these reasons that the NMR analyses are further supported with X-ray, HPLC and HRMS analyses. HRMS (MALDI) calc for $[C_{13}H_{11}O_2N_3S_2 + Na]^+$ 328.01849, found 328.01880. Additionally, samples used in this study were subjected to elemental analysis to confirm >95% purity: Anal. Calcd for $C_{13}H_{11}O_2N_3S_2$: C, 51.13; H, 3.63, N, 13.76. Found C, 50.80; H, 3.52; N, 13.67.

1.2. 1,6′-DP: Crude 1,6′-DP was obtained from (Aevis Bio Inc., Daejeon, Republic of Korea). In general, Pomalidomide was combined with Lawesson's Reagent and heated in refluxing toluene. The reaction progress was monitored, and the 1,6′-DP isomer was the major dithioanted product within the crude material. Upon receipt of the crude material, further purification and characterization was performed as per Scerba et al.[1]. Specifically, crude material was purified by prep HPLC to give an orange powder. ^{1}H NMR (400 MHz, DMSO-d_6) δ 12.64 (s, 1H), 7.47 (t, J = 7.2 Hz, 1H), 7.13-7.06 (m, 2H), 6.60 (bs, 2H), 5.86-5.41 (bm, 1H*), 3.24 – 2.83 (b, 2.3H*), 2.01 (bs, 1H). Note that the starred * signals are very broad and proved difficult to resolve even after increasing relaxation delays and scans. It is for these reasons that these NMR analyses are further supported with X-ray, HPLC and HRMS analyses. HRMS (ESI) calc for $[C_{13}H_{11}O_2N_3S_2 + Na]^+$ 328.01849, found 328.01775. Additionally, samples used in this study were subjected to elemental analysis to confirm >95% purity: Anal. Calcd for $C_{13}H_{11}O_2N_3S_2$: C, 51.13; H, 3.63, N, 13.76. Found C, 50.88; H, 3.49; N, 13.74.

2. FDA Required Drug Development Regulatory Studies

2.1. In Vitro Mammalian Chromosome Aberration Test

2.1.1. Overview: The potential ability of 1,6′- and 3,6′-dithiopomalidomide (1,6′-DP and 3′6′-DP, respectively) to damage mammalian chromosomal structure was evaluated *in vitro* using human peripheral blood lymphocytes (HPBLs). HPBLs were cultured with three concentrations of each test compound under three different experimental paradigms, and the actions on chromosome structure were then assessed. The following chromosome alterations were examined: gaps, breaks (including deletions and/or displacements) and exchanges. To additionally investigate the potential of Phase 1 metabolism on

the test compounds to generate genotoxic metabolites, the cells were incubated with Aroclor 1254 induced rat liver S9 extracts in the presence of the drugs.

2.1.2. Methods: Briefly, HPBLs were cultured with test compounds (concentrations: 125, 250 and 500 μg/ml) in the presence or absence of metabolic activation (S9 extracts). HPBLs were cultured under three different experimental paradigms: Experiment 1: Cells were incubated in the presence of the compounds and rat liver S9 extract for 3 hr. Then, after a series of washes, the cells were cultured with fresh media for an additional 18 to 19 hr. Experiment 2: Cells/compounds were not co-incubated with rat liver S9 extracts; otherwise HPBLs were treated identically as described in Experiment 1. Likewise, after the 3 hr incubation the test compounds were removed, and the cells were washed and cultured for an additional 18 to 19 hr. Experiment 3: HPBLs were cultured in the presence of the test compounds for 22 hr. Various controls were incorporated into the studies, HPBLs were challenged with the compound drug vehicle (dimethyl sulfoxide (DMSO)) and cells were challenged with chemicals known to induce structural alterations to mammalian chromosomes (Experiment 1: cyclophosphamide monohydrate (CPA: 14 μg/ml); Experiment 3: ethyl methanesulfonate (EMS: 600 μg/ml) to provide positive controls. Following the different exposure times, cells were arrested in a metaphase-like state by use of colchicine, and samples were re-suspended in 0.56% KCl to induce cellular swelling. Thereafter, cells were washed with fixative (cold methanol/acetic acid, 3:1 v/v), centrifuged and re-suspended in fresh fixative generating a cell slurry. This was placed on glass slides that were dried, stained with Giemsa stain, and then xylene mounted with dibutyl phthalate in xylene. These slides were used to observe the effects of the test compounds on the cell chromosomes.

2.1.3. Results: 1,6′- and 3,6′-DP at 250 and 500 μg/ml proved to be toxic to HPBLs; hence all chromosomal aberration data is derived from HPBLs challenged with drugs at 125 μg/ml. The drug vehicle action on cells was determined to be in line with historical data. The effects of both 1,6′- and 3,6′-DP (125 μg/ml, with S9 extract-induced metabolism of the compounds) on the HPBLs were observed to be similar to the drug vehicle treated cells (**Suppl Figure S1A**). The positive control, CPA, induced chromosomal damage by the formation of gaps, breaks and exchanges in the HPBL cell DNA. HPBLs incubated with non-metabolized compounds displayed identical responses to those seen for the cells incubated in the presence of rat liver S9 extracts. A more chronic exposure of HPBLs with the test compounds for 22 hr illustrated similar results. In contrast to this, the presence of EMS caused the formation of gaps, breaks and exchanges in the HPBL chromosomes (**Suppl Figure S1B**). In synopsis, these studies demonstrated that 1,6′- and 3,6′-DP do not induce mammalian chromosome aberration at 125 μg/ml.

Suppl Figure S1

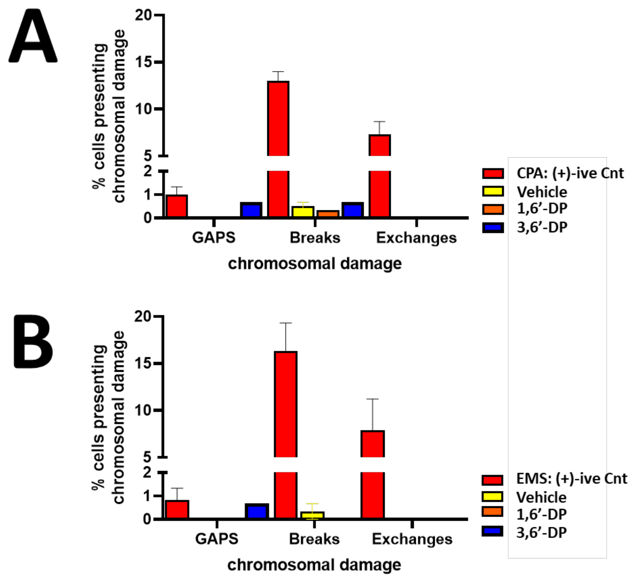

Suppl Figure S1. Effects of 1,6′- and 3,6′-dithiopomalidomide on mammalian HPBL chromosome structure. **(A)** illustrates the percentage of HPBLs displaying chromosomal abnormalities including gaps, break and exchanges, when compounds were co-incubated in the presence of rat liver S9 fraction extracts. The effects of both 1,6′- and 3,6′-dithiopomalidomide (1,6′- and 3,6′-DP: 125 μg/ml) were observed to be similar to that of the drug vehicle (DMSO). In contrast, the mutagenic positive control ((+)-ive Cnt) cyclophosphamide (CPA: 14 μg/ml) effectively induced gaps, breaks and exchanges in the chromosome DNA. **(B)** demonstrates that the longer-term incubation of HPBL cells with either vehicle or test compounds in the absence of metabolic activation were similar. In contrast, treatment of the cells with the mutagen ethyl methanesulfonate (EMS: 600 μg/ml) induced gaps, breaks and exchanges in chromosomal DNA. HPBL cells numbers were evaluated in the studies ranged from 150 to 300 cells per treatment.

2.2. AMES Fluctuation test

2.2.1. Overview: 1,6′- and 3,6′-DP were screened in a miniaturized version of the AMES Fluctuation test for mutagenicity [2]. This assessment follows the standard AMES assay, but is performed in a 96 well plate format. Evaluation of the compounds was undertaken in the absence of metabolic activation and, to mimic Phase 1 metabolism, in the presence of metabolic activity by inclusion of Aroclor 1254 activated rat liver homogenate S9 fraction (S9 fraction plus NADP, glucose-6-phosphate, $MgCl_2$ and KCl in phosphate buffered saline). All four concentrations (5, 10, 50 and 100 μM) of the two compounds (1,6′ and 3,6′-DP) were screened in the fluctuation tests. DMSO was used as the drug vehicle control. Both drugs were screened in five different genetically modified bacterial strains (*Salmonella typhimurium* (TA98, TA100, TA1535 and TA1537) and *Escherichia coli* (WP2uvrA)). The bacterial strains TA98, TA100, TA1535 and TA1537 are dependent on the presence of the amino acid 'histidine' whereas the strain WP2uvrA requires the presence of 'tryptophan' in the growth media for bacterial cell survival. The strains are engineered such that in the event that a test chemical induce mutations (frameshift and/or base substitution mutations) within the bacterial DNA, the strains would no longer have the requirement for the presence of histidine or tryptophan in the growth media to support survival. Hence, in the presence of mutagenic chemicals, the strains would be able to unrestrictedly grow. To validate the mutagenicity assays, each bacterial strain was exposed to a test chemical known to induce mutagenicity in that strain. In the presence of Aroclor 1254 activated rat liver homogenate S9 fraction; 2-aminoanthracene (0.2 μg/ml) was

used as the positive control for TA98, TA100, TA1535, TA1437 and WP2uvrA. In the absence of Aroclor 1254 activated rat liver homogenate S9 fraction; 2-Nitrofluorene (1 μg/ml) was used as the positive control for TA98, sodium azide (1 μg/ml) as the positive control for TA100 and TA1535, 9-aminoacridine (1 μg/ml) as the positive control for TA1537, and 4-nitroquinoline-1-oxide (1 μg/ml) as the positive control for WP2uvrA. Biological readout for evidence of test compound induced-mutagenicity was assessed by use of a pH dependent dye in the growth media (bromocresol purple). When wells contain actively growing cells, the growth media pH becomes acidic and this changes the dye color from blue-green to yellow. The presence of yellow wells, thus, indicates mutagenesis. In contrast, blue-green wells indicate no evidence of mutagenesis.

2.2.2. Performance of the assay: One 96 well plate was utilized for each test condition. The test compounds were prepared and mixed with bacterial growth media (containing low levels of histidine/tryptophan, in the absence or presence of Aroclor 1254 activated rat liver homogenate S9 fraction). In the absence of S9 fraction extracts, equal aliquots of growth media containing the different bacterial strains ± test compounds were added to 96 well plates that were then incubated for 3 days at 37°C in a humidified chamber. In the presence of the S9 fraction extracts, bacteria were pre-incubated with the test compounds for 16 to 18 hr. Thereafter, growth media (containing low levels of histidine/tryptophan) was added and the 96 well plates were grown for 3 days in a humidified chamber at 37°C. For both assessments (absence/presence of metabolic activation) the numbers of yellow or blue-green wells were counted, and the data presented as numbers of yellow/green or blue wells per 96 well plate.

2.2.3. Results: Low numbers of actively growing bacterial strains were observed in the drug vehicle treated cells across the different bacterial strains. The levels of actively growing bacteria in the 1,6′- and 3,6′DP treated wells were observed to be similar to those of the drug vehicle controls. There were no drug dose-dependent elevations in the numbers of positive wells for either test compound (**Suppl Figure S2, Panel A**). Additionally, these effects were not influenced by prior metabolic activation of the compounds with the rat live S9 fraction (**Suppl Figure S2, Panel B**). To confirm the validity of the assay system, each of the bacterial strains responded strongly to the appropriate positive control, mutation-inducing chemical treatments. These effects were not altered by the presence or absence of the rat liver S9 fraction (**Suppl Figure S2, A/B**).

Suppl Figure S2.

Suppl Figure 2 Panel A

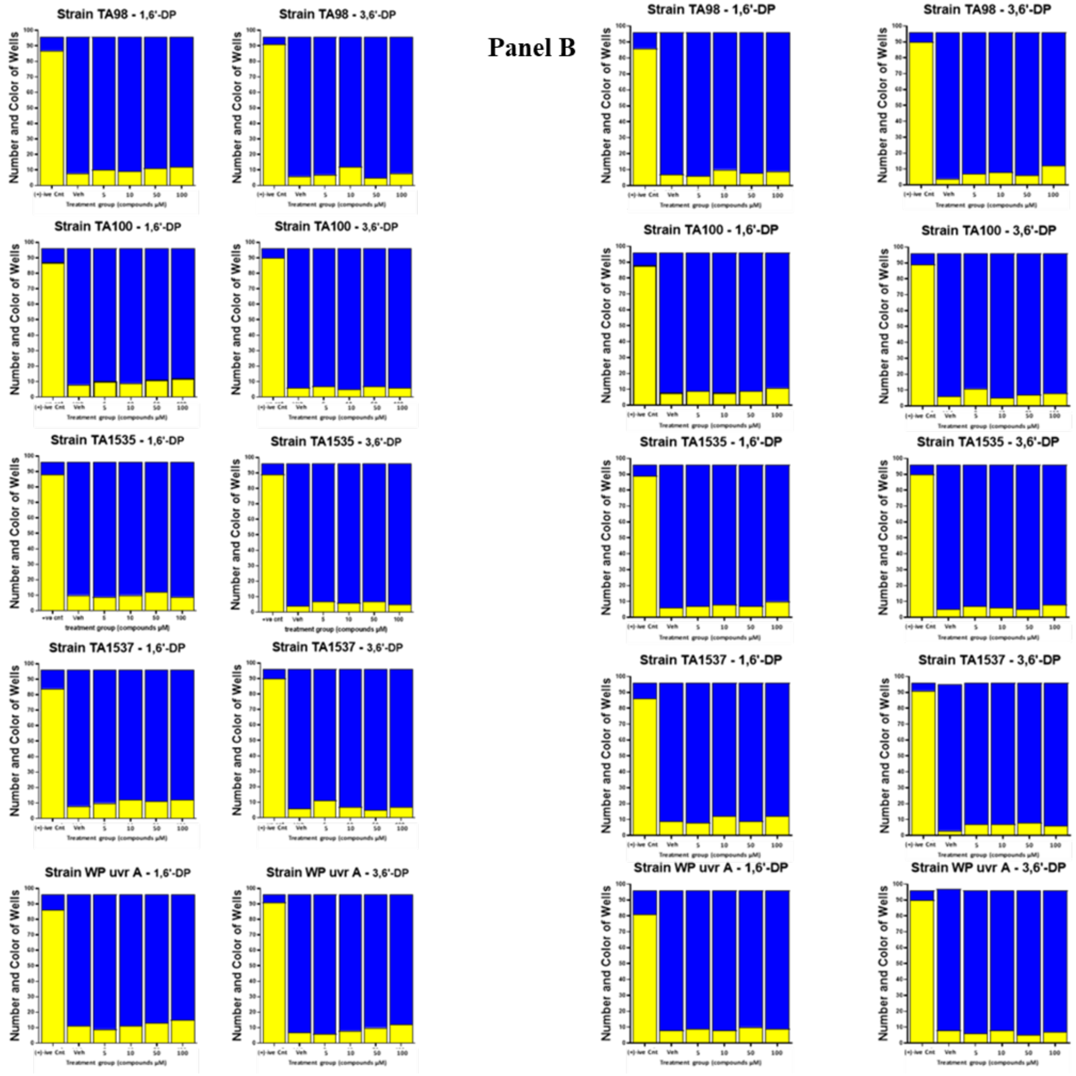

Suppl Figure S2. Assessment of the potential mutagenic effects of 1,6′-dithiopomalidomide and 3,6′-dithiopomalidomide on bacterial DNA. **(A)** In the absence of test compound metabolism, the effects of the different treatment conditions on bacterial DNA mutations are shown. Bacterial cell growths are presented as yellow bars, whereas absence of bacterial cell growth is presented as blue bars. Treatment of the different bacterial strains with drug vehicle (Veh: DMSO) induced low numbers of actively growing cultures (yellow bars) and high numbers of wells where cells did not grow (blue bars). The effects of the different concentrations of the test compounds 1,6′- and 3,6′-DP, on bacterial cell growth were similar to Veh-treated cells. In contrast, positive controls ((+)-Cnt) for mutations induced high numbers of actively growing bacteria (yellow bars). **(B)** In the presence of test compound metabolism, the levels of bacterial cell growth were identical to the cell growth found in the absence metabolism seen in Panel A. Each experimental condition was assessed in one 96 well plate.

2.4. Assessment of potential drug interaction with hERG channel function

2.4.1. Overview: The hERG (human Ether-a-go-go Related Gene) assay is an essential assessment made with drug candidates to study for possible cardiac toxicity. The hERG channel is a potassium channel (rapid component of the delayed rectifier potassium current, IKr) and is partly responsible for the repolarization of cardiac cells. In the event that a drug candidate interferes with the normal function of

the hERG channel, a prolonged repolarization phase may result that provides the electrophysiological substrates for life threatening cardiac arrhythmias. The hERG channel can be stably expressed in cell lines (i.e., human embryonic kidney 293 cells; (HEK 293 cells)), thereby allowing characterization of IKr currents in the presence/absence of drugs by use of electrophysiological methods.

2.4.2. Methods: The effects of 1,6′- and 3,6′-DP at four concentrations (1, 3, 10 and 30 μM) were assessed on hERG-HEK263 cells. In brief, drug stocks were prepared in 100% DMSO; the drug vehicle and drug stocks were diluted in hERG-HEK293 cell buffer solution. Once stable patch-clamped seals were generated, the cells were washed and then baseline electrophysiological recordings, in the drug vehicle, were obtained. After approximately 5 min of stable recordings, the effects of 1,6′- and 3,6′-DP, in cumulative concentrations, on IKr channel conductance were assessed. After the assessment of 1,6′- and 3,6′-DP at a concentration of 30 μM, the cells were challenged with the drug propafenone (10 μM), which was used as a positive control for the inhibition of the IKr channel conductance.

2.4.3. Results: The effects of the highest concentration of 1,6′-DP (30 μM) on hERG channel conductance were observed to be less than 5% of the control channel conductance. Lower 1,6′-DP concentrations resulted in negligible inhibition. Treatment of cardiomyocytes with 3,6′-DP (30 μM) resulted in a larger effect on hERG channel conductance, and resulted in a 27% reduction compared to the control channel conductance level. The antiarrhythmic drug propafenone was used as a positive control in the light of its known potency to inhibit hERG potassium channels. The inhibitory action of propafenone (10 μM) on hERG channel conductance was markedly larger than that observed for either 1,6′- or 3,6′-DP, inducing an IKr conductance inhibition of approximately 85% (**Suppl Figure S3).**

Suppl Figure S3 – hERG channel conductance

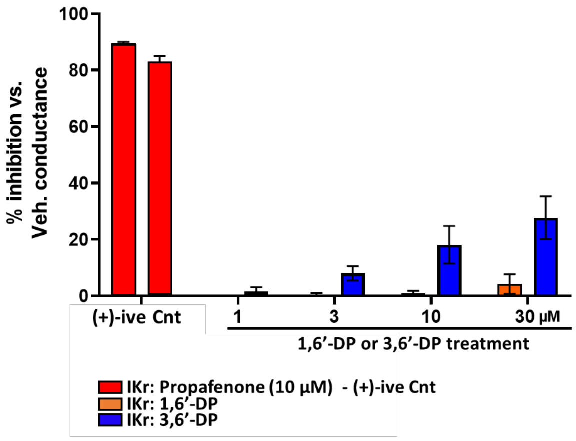

Suppl Figure S3. Effects of 1,6′-dithiopomalidomide and 3,6′-dithiopomalidomide on human Ether-a-go-go Related Gene (hERG) channel activity. The effects of the test compounds 1,6′- and 3,6′-dithiopomalidomide (1,6′- and 3,6′-DP) on the activity of the hERG channel conductance are presented as the percentage inhibition of the channel activity. The highest evaluated concentration of 1,6′-DP caused a small reduction in ion channel conductance (~5%), and 3,6′-DP a slightly larger (~27%) inhibition. Both actions were markedly smaller than that of the positive control, propafenone (10 μM: 80-90% inhibition). The data presented were obtained from assessments of test compounds made on different days with 2 to 5 cells.

3. In vivo Stroke Studies

3.1. MCAo Methodology:

3.1.1. MCAo procedure: Male Sprague Dawley rats, weighing 175-200 g, were obtained from BioLASCO Taiwan for ischemic stroke studies. All animals were maintained under a 12/12-hr day/night cycle and were housed at an ambient temperature with free access to food and water at the Experimental Animal Center of Taipei Medical University. All animal study methods were carried out in accordance with the National Institutes of Health (DHEW publication 85–23, revised, 1996). Animal numbers for each assessment group and experimental measures were selected based upon our prior studies [3]. Their use in research was approved by the Animal Care and Use Committee of the National Defense Medical Center (NDMC), Taipei, Taiwan (protocol No. IACUC-20-043).

Rat transient MCAo model and drug treatment: The MCAo was performed as described previously [4, 5]. Briefly, the rats were anesthetized using i.p. administered Zoletil + Xylazine (Rompun) 25mg/kg + 2.332 mg/kg. The bilateral common carotids were then ligated with non-traumatic arterial clips. A craniotomy of approximately 2 × 2 mm^2 was applied to the right squamosal bone. Cerebral ischemia was induced by ligation of the right MCA with a 10-O suture, thereby inducing MCAo for 60 min. The ligature and clips were removed after 60 min ischemia to generate re-perfusional injury. Core body temperature was monitored with a thermistor probe and maintained at 37 °C with a heating pad throughout anesthesia. After recovery from the anesthesia, body temperature was maintained at 37 °C using a temperature-controlled incubator. Immediately after the recovery from anesthesia, an elevated body swing test was used to evaluate the success of MCAo surgery. All animals used for this study demonstrated prominent motor bias contralateral to the lesion side, and animal numbers utilized were based on the variance of data and differences between treatment and control groups evident in both our prior studies and those of others.[5-7]

The rats were divided into five experimental groups: sham (without MCAo) and, for those with MCAo, randomly (computer generated) between vehicle MCAo, 3,6′-DP, 1,6′-DP, and Pomalidomide MCAo groups. Following the period of 60 min ligation and 30 min reperfusion, the groups received i.p. injections of vehicle 3,6′-DP, 1,6′-DP, or Pomalidomide, respectively (Pomalidomide 20 mg/kg; 3,6′-and 1,6′-DP 21.25 mg/kg (i.e., equimolar doses)). Vehicle was DMSO.

3.2. Assessment of the injured cerebral lesion area and neurological deficit scores:

Infarct size was measured based on Shen et al. [5] at 24 hr after MCAo, and was performed by an observer blinded to the treatment groups. Brains were quickly removed and immersed into cold saline for 5 min. Coronal slices were dissected into 2 mm from the frontal tips. Sections were immersed in 2% TTC at 37°C for 10 min and subsequently fixed in a 5% formaldehyde solution. The brain infarct volume was calculated as a percentage zone of the coronal section in the infarcted hemisphere.[5, 7] Lateral movements and turning of the body were assessed using the body asymmetry test, as detailed by Borlongan and Sanberg [6,7]. Specifically, rats were lifted from their tails and maintained at 20 cm above the examination table. The frequency of initial turning of the upper body contralateral to the ischemic side was calculated in 20 subsequent trials. The maximum impairment in body swing in MCAo rats is 20 contralateral turns/20 trials, in which animals demonstrate asymmetric behavior ipsilateral to the MCAo.[6, 7] An uninjured animal would show a value of 10 (i.e., an equal number of left and right turns).

3.3. Cytokine evaluation in MCAo challenged rats:
Whole blood samples (0.5 ml) were collected 24 hr following MCAo, and plasma was isolated for quantification of post-treatment cytokine levels across all groups. A MILLIPLEX® (Milliplex MAP Rat Cytokine/Chemokine Kit; Millipore, Billerica, MA, USA) multiplex panel was used to quantify rat plasma cytokine levels. The commercially available multiple bead-based kit was applied to quantify TNF-α, IL-1β and IL-10 in line with the manufacturer's protocol. Samples were centrifuged (15000 rpm, 4℃, 30 min) to remove any particulate material. Briefly, 25 μL of plasma was incubated with fluorescently-labeled capture antibody-coated beads in a 96-well filter bottomed plate on a plate shaker for 2 hr at room temperature. Biotinylated detection antibodies were then added and incubated for 60 min at room temperature with shaking. The reaction mixture was detected by the addition of streptavidin-phycoerythrin. Beads were re-suspended in sheath fluid for 5 min on a plate shaker. Plates were read on a Luminex® analyzer (MAGPIX®) (Millipore, Billerica, MA, USA) and analyzed using xPONENT® MILLIPLEX® Analyst 5.1 software (Millipore, Billerica, MA, USA) with a five-parameter model used to calculate final concentration values (expressed in pg/mL).

4. Cereblon Studies

4.1. Cereblon binding and neo-substrate studies in human cell cultures:
Quantitative evaluation of cereblon binding was performed by utilizing bead-based AlphaScreen technology. (BPS Bioscience #79770). Pomalidomide, 3,6'-DP or 1,6'-DP was incubated at concentrations between 0.01 and 100 μM with reaction mixtures that contained cereblon/DDB1-CUL4A-Rbx1 (Cullin 4a-Ring-box protein 1) complex (12.5 ng) and bromodomain-containing protein 3 (BRD3) (6.25 ng) in an Optiplate 384-well microplate (PerkinElmer, #6007290). After 30 min incubation with shaking, AlphaLISA anti-FLAG Acceptor and Alpha Glutathione Donor beads (PerkinElmer) were then added and, thereafter, were incubated (60 min, RT, for each of the added agents). After this, alpha-counts were recorded on a Synergy Neo2 microplate reader (BioTek). Relative activity was determined as the highest value, which was established as 100%, and the lowest value was defined as 0% following appropriate subtraction of the 'blank value' from all readings.

The actions of Pomalidomide, 3,6'-DP and 1,6'-DP on the neo-substrates Aiolos and Ikaros were characterized in human MM1.S (multiple myeloma) cells (ATCC, Manassas, VA, USA). MM.1S cells were grown in RPMI media that was supplemented with 10% FBS, penicillin 100U/ml and streptomycin 100μg/ml, and were maintained at 37°C and 5% CO_2. MM1.S cells were treated with 0.01, 0.1, 1μM of Pomalidomide, 3,6'-DP or 1,6'-DP for 24hr. The actions of the same drugs on levels of the neo-substrate SALL4 were evaluated in H9 hESC (human embryonic stem) cell lines. These were obtained from WiCell Research Institute (#WA09; Madison, WI, USA), and cultured on growth factor reduced matrigel coated dishes in mTeSR1 media (STEMCELL Technologies, Vancouver, Canada), supplemented with 5ng/ml bFGF, penicillin 100U/ml and streptomycin 100μg/ml, and maintained at 37°C and 5% CO_2. Cell lysates derived from control and drug treatments of MM1.S and H9 cells were ultimately prepared for Western blot analysis, as described previously [8].

For Western blot studies, total proteins were extracted with RIPA buffer (ThermoFisher Scientific, Waltham, MA, USA) containing Halt Protease Inhibitor Cocktail (ThermoFisher Scientific). Thereafter,

proteins were separated by gel electrophoresis, and transferred to polyvinylidene difluoride (PVDF) membranes (ThermFisher Scientific), as described previously [9]. The primary antibodies probed included: (i) anti-Aiolos antibody (cat#15103; 1:1000 dilution; Cell Signaling Technology), (ii) anti-Ikaros antibody (cat#9034; 1:1000 dilution; Cell Signaling Technology, Danvers, MA, USA), (iii) anti-SALL4 antibody (SC101147; 1:1,000; Santa Cruz Biotechnology, Dallas, TX, USA), (iv) anti-β-actin antibody (CST #3700; 1:5,000; Cell Signaling Technology) and (v) GAPDH antibody (cat#ab8245; 1:5000 dilution; Abcam). After an overnight incubation (4°C), HRP conjugated secondary antibodies were used that included (i) Goat anti-rabbit IgG (ThermoFisher Scientific), and (ii) Goat anti-mouse IgG. GAPDH was used as an internal control (house-keeping protein), against which SALL4, Aiolos and Ikaros protein expression levels were compared. Antigen–antibody complexes were detected using enhanced chemiluminescence (iBright CL1500, ThermoFisher Scientific).

5. Anti-inflammatory Studies

5.1. In vivo lipopolysaccharide (LPS) inflammatory challenge:

Male Fischer 344 rats (Charles River Laboratories, Wilmington, MA, USA) (approx. 150 g weight) were housed in the Animal Facility of the Intramural Research Program, National Institute on Aging, NIH, Baltimore, MD, USA, in accord with National Institutes of Health DHEW publication 85–23, revised, 1996 and approved Animal Care and Use Committee protocol No. 331-TGB-2018 and 415-TGB-2021. These rats were randomly assigned across groups, and then administered vehicle, or either 1,6′-DP or 3,6′-DP (29.5 mg/kg, i.p.), 60 min prior to either administration of LPS (1 mg/kg, Sigma, St Louis, MO, *E.coli* O55:B5 in saline (0.9%), 0.1 ml/kg i.p. or vehicle. The drug dose was selected to be equimolar to that of thalidomide (25 mg/kg), which has been demonstrated to be well-tolerated in prior rodent studies and of translational relevance to humans. At 4 hr following LPS or vehicle, animals were euthanized, and plasma and brain (hippocampus) tissue samples were quickly harvested on wet ice and immediately stored at -80°C. Brain samples were later sonicated in a Tris based lysis buffer (Mesoscale Discovery) with protease/phosphatase inhibitors (Halt™ Protease and Phosphatase Inhibitor Cocktail, ThermoFisher Scientific, diluted to 3X). Thereafter, brain samples were centrifuged (10,000 g, 10 min, 4°C), and protein concentrations were determined by Bicinchoninic acid assay (BCA, ThermoFisher Scientific). Finally, an ELISA for TNF-α, IL-6, IL-1β, and IL-10 was later performed following the manufacturer's protocol (Mesoscale Discovery).

5.2. Cellular inflammatory and survival studies

5.2.1. 1,6′-DP and 3,6′-DP activity in primary dopaminergic neurons and microglia challenged with α-synuclein: Rat dopaminergic neurons and microglia were separated, and maintained in culture. On day 7 in culture, cells were pre-incubated for 1 hr with culture medium freshly spiked with known concentrations of 1,6′-DP, 3,6′-DP or veh (0 to 30 μM), and were then exposed to α-synuclein (250 nM α-synuclein, 72 hr, n=6 per group: human recombinant α-synuclein 1-140 aa from rPetide, Watkinsville, GA), which in a prior study was determined to contain <1.3 U endotoxin /mg of peptide, a concentration incapable of producing any significant effects in terms of neurotoxicity or induction of ROS production. α-synuclein was previously prepared as a 4 mM solution and slowly shaken at 37° C for 72 hr

in the dark to induce oligomerization. Thereafter, cell culture supernatants were removed and frozen for later assays, and cells were washed in physiological buffered saline (PBS).
Quantification of the following was undertaken: (i) dopaminergic neuron survival (as determined by counting the number of tyrosine hydroxylase (TH)-positive neurons across conditions, in comparison to the control (Veh) condition – i.e., without α-synuclein challenge). (ii) The total neurite network of the dopaminergic neurons (as determined from the length of TH-positive neurites) (iii) Total microglia activation (as determined from the area of microglial cells, μm^2 of OX-41 staining). (iv) TNF-α generation was determined by quantifying the level of TNF-α protein in cell culture media samples by ELISA (rat TNF-α ELISA kit, Abcam, ab46070).

5.2.2. Drug assessment of anti-inflammatory actions in LPS activated RAW 264.7 cells: Cells were seeded into 24 well plates (PerkinElmer Black Visiplate ™TC # 1450-605 at a density of 250 x 10^3 cells per well. Twenty-four hours after seeding, the seeding media was replaced with fresh media. Two hours later the cells were exposed to a range of concentrations of drug test-compound(s) dissolved in 100% tissue culture grade dimethyl sulfoxide (DMSO, Sigma #D2650). The effects of each concentration of drug were assessed in 4 wells per concentration in the 24 well plate (n = 4). On each plate, one set of wells were assigned as drug-vehicle control (i.e., DMSO+LPS); one test compound was assessed on one 24 well plate. The drug concentrations used for each test agent were 600 nM, 1, 10, 30 and 60 μM. One hour after the drugs were added the cells were challenged with lipopolysaccharide (LPS, Sigma, serotype 055:B5) at a final concentration of 60 ng/ml. Twenty to twenty-four hours after the cells were challenged with LPS, the culture media was collected and utilized for the measurement of markers of inflammation.

5.2.3. RAW 264.7 cell viability evaluation: RAW 264.7 cell viability was determined by use of a fluorescent cell viability assay, the CellTiter-Blue Cell Viability assay (Cat # G8081, Promega, Madison. WI). The plate was read with an excitation at 560 nm λ and the emission was read at 590 nm λ using an Infinite M200 PRO plate reader (TECAN, USA). The relative fluorescence unit (RFU) levels from the different drug treatments were compared to those of the control wells (DMSO+LPS controls) and the RFUs were then converted to relative percentage change of control levels for each compound.

5.2.4. Nitrite ion detection in drug treated RAW 267.4 cell culture media: Levels of nitrite ion (NO_2^-) were quantified by the Nitrate/Nitrite Fluorometric Assay Kit (Abnova, Cat # KA1344) as a surrogate of NO levels, as nitrite is a stable non-volatile breakdown product of NO. RFUs/NO_2^- levels were assessed as follows. The plate was read with an excitation at 362 nm λ and an emission at 430 nm λ (Infinite M200 PRO plate reader, TECAN, USA). The raw RFU data for the standards and unknowns were used to calculate the NO_2^- levels (μM) using linear regression analysis in GraphPad Prism 9.2.0 (GraphPad Prism). NO_2^- levels were then converted to relative percentage change of control levels for each set of drug treatments (DMSO+LPS controls).

5.2.5. Enzyme-linked immunosorbent assay for TNF-α protein: Media TNF-α levels were measured by use of the Biolegend ELISA MAX Set Delux ELISA (#430904). A day prior to performing the assay a 96 well plate was coated with a capture antibody directed against TNF-α. The absorbance was read 450 nm λ

and for background subtractions at 570 nm λ, on a SPECTRAmax PLUS plate reader. The absorbances were used to generate a TNF-α protein standard curve, and the protein levels in the unknown samples were then determined using SoftMax Pro V5, Molecular Devices. The TNF-α levels were thereafter converted to relative percentage change of control for each set of drug treatments (DMSO+LPS controls).

6. References

1. Scerba M.T.;. Siegler M.A.; Greig N.H. Thionation of aminophthalimide hindered carbonyl groups and application to the synthesis of 3,6′-dithionated Pomalidomides. Synlett **2021**, 32**,** 917-922.
2. Ames B.N.; Gurney E.G.; Miller J.A.; Bartsch H. Carcinogens as frameshift mutagens: metabolites and derivatives of 2-acetylaminofluorene and other aromatic amine carcinogens. *Proc Natl Acad Sci U S A* **1972**, *69*, 3128-32.
3. Tsai Y.R.; Chang C.F.; Lai J.H.; et al. Pomalidomide ameliorates H_2O_2-induced oxidative stress injury and cell death in rat primary cortical neuronal cultures by inducing anti-oxidative and anti-apoptosis effects. *Int J Mol Sci*. **2018**, *19*, 3252
4. Chen S.T.; Hsu C.Y.; Hogan E.L.; Maricq H.; Balentine J.D. A model of focal ischemic stroke in the rat: Reproducible extensive cortical infarction. *Stroke*. **1986**, 17, 738-743
5. Shen H.; Luo Y.; Kuo C.C.; Deng X.; Chang C.F.; Harvey B.K.; et al. 9-cis-retinoic acid reduces ischemic brain injury in rodents via bone morphogenetic protein. *J Neurosci Res*. **2009**, *87*, 545-555
6. Borlongan C.V.; Sanberg P.R. Elevated body swing test: A new behavioral parameter for rats with 6-hydroxydopamine-induced hemiparkinsonism. *J Neurosci*. **1995**, *15*, 5372-5378
7. Borlongan CV, Hida H, Nishino H. Early assessment of motor dysfunctions aids in successful occlusion of the middle cerebral artery. *Neuroreport*. **1998**, *9*, 3615-3621
8. Huang P.S.; Tsai P.Y.; Yang L.Y.; et al. 3,6'-dithiopomalidomide ameliorates hippocampal neurodegeneration, microgliosis and astrogliosis and improves cognitive behaviors in rats with a moderate traumatic brain injury. *Int J Mol Sci*. **2021**, *22*, 8276
9. Lin C.T.; Lecca D.; Yang L.Y.; et al. 3,6'-dithiopomalidomide reduces neural loss, inflammation, behavioral deficits in brain injury and microglial activation. *Elife*. **2020**, *9*, e54726
